# Development and validation of a non-chromatographic method for mercury and methylmercury in finfish using thermal decomposition gold amalgamation atomic absorption spectrophotometry (TDA-AAS) and salting-out assisted liquid–liquid extraction (SALLE)

**DOI:** 10.1007/s00216-025-05989-8

**Published:** 2025-07-16

**Authors:** Jake A. Carter, Charles A. Barber, Mesay M. Wolle, Patrick J. Gray

**Affiliations:** Human Foods Program, Office of Laboratory Operations & Applied Science, US Food & Drug Administration, 5001 Campus Drive, College Park, MD 20740 USA

**Keywords:** Methylmercury, Mercury, Finfish, SALLE, TDA-AAS

## Abstract

Accurate technologies and methods are needed to monitor both total and methylmercury in the marine food supply. Thermal decomposition gold amalgamation atomic absorption spectrophotometry (TDA-AAS) is an efficient and cost-effective technique for measuring low levels of total mercury requiring no sample preparation. Measuring methylmercury with TDA-AAS requires isolating methylmercury from the matrix and other mercury species prior to detection. We developed a method that uses ethyl acetate instead of a legacy non-polar solvent, toluene. Toluene is a potentially hazardous and problematic solvent whereas ethyl acetate is greener and safer. Additionally, the salting-out assisted liquid–liquid extraction (SALLE) approach with ethyl acetate avoids emulsion formation throughout extraction. We describe method development and validation of a SALLE and TDA-AAS detection for methylmercury in finfish. From 10 reference materials, our method recovered 80–118% of total or methylmercury with *Z* scores ranging from −1.98 to 2.75 (*n* = 184). The LOD and LOQ of the method for methylmercury were 3.8 and 27 ng/g, respectively. From extraction to detection, accurate results were obtained from a sample in less than 2 h for both total mercury and methylmercury.

**Graphical Abstract:**

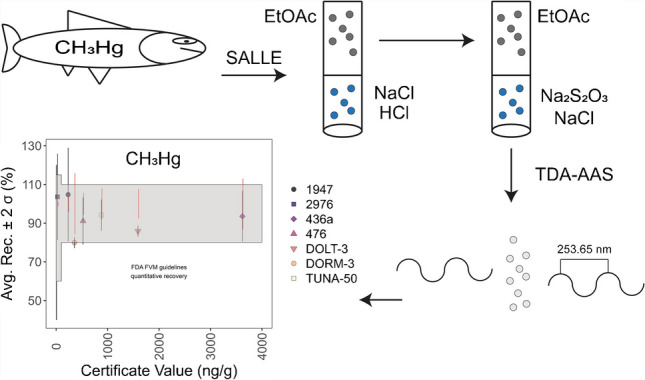

**Supplementary Information:**

The online version contains supplementary material available at 10.1007/s00216-025-05989-8.

## Introduction

Methylmercury is the most common and potentially harmful form of mercury found in seafood [1]. Human dietary exposure to mercury and methylmercury occurs mainly from fish and seafood consumption [1, 2]. The marine environmental load of mercury depends on factors including natural geological makeup and anthropological inputs from human activity and industrial runoff. These sources affect the levels of mercury found in seafood [1, 3]. The persistence of this pollutant in the marine environment, where environmental mercury is converted to and bioconcentrated as methylmercury up the food chain, warrants monitoring in the food supply [1, 3, 4]. Therefore, accurate techniques and methods are needed to measure mercury and methylmercury in seafood, specifically finfish.

Current method approaches commonly use inductively coupled plasma mass spectrometry (ICP-MS), which is a sensitive, accurate, and multielement technique capable of measuring trace levels of mercury in foods including finfish [5]. ICP-MS, however, requires foods to be decomposed and liquified prior to analysis [6, 7], which requires the use of corrosive acids and additional sample preparation instrumentation, and is time and reagent consuming [8]. Thermal decomposition gold amalgamation atomic absorption spectrophotometry (TDA-AAS) is another technique capable of measuring mercury in food. The main advantage of TDA-AAS over ICP-MS is that no sample preparation is required for determining total mercury, where a TDA-AAS instrument can provide an accurate result in less than 10 min [9, 10]. TDA-AAS and ICP-MS are both atom-based techniques that are blind to the molecular structure of elements. As a result, analyzing finfish for methylmercury by ICP-MS requires a chromatography system such as high-performance liquid chromatography (HPLC) to separate mercury species on a column prior to ionization in the ICP [11]. Similarly, TDA-AAS detection of methylmercury requires prior separation of methylmercury from the matrix and other mercury species. Taking advantage of the polarity of mercury species in acidic halide solutions allows for separation of methylmercury prior to TDA-AAS detection [12, 13].

A legacy method from the 1960s, featuring acidic halide extractions of methylmercury from fish, required benzene and large amounts of sample and reagents [14, 15]. This method was adapted for cold vapor atomic absorption spectrophotometry by Zanicchi et al. who replaced benzene with toluene [16] Scerbo and Barghigiani modified and validated this method for methylmercury detection by TDA-AAS, which was further validated by Maggi et al. through an interlaboratory validation exercise [12, 17, 18]. Azemard and Vassileva improved this method further by reducing the extraction time and the amount of generated waste [19].

Toluene is the most common solvent reported in liquid–liquid extraction methods for methylmercury detection by TDA-AAS [12, 17, 20]. It is a non-polar solvent and less hazardous than benzene, but it is still a potentially problematic solvent due to its health effects and impact on the environment. Solvent selection guides do not recommend routine use of toluene [21–24]. Salt-assisted liquid–liquid extraction (SALLE) is an extraction strategy that allows for emulsion-free phase separation of water-miscible solvents from water and the extraction of non-polar and polar analytes [25]. SALLE was first introduced to separate acetone from aqueous solutions to enhance the sensitivity of flame atomic absorption spectrometry [26]. However, the most used and studied solvent in a SALLE system is acetonitrile. Various solvents and salts can be used depending on the polarity of the analyte and required phase separation [25, 27–29].

We describe method development and validation of a non-chromatographic method for the measurement of total mercury and methylmercury in finfish using SALLE and TDA-AAS. The SALLE-TDA-AAS approach is comparable in accuracy to traditional chromatographic methods, requiring significantly less time and money. We replaced toluene with ethyl acetate and sodium chloride (NaCl) in a SALLE extraction of methylmercury prior to detection by TDA-AAS. Using ethyl acetate instead of toluene leads to a safer and greener method, relying on a “recommended” instead of a “problematic” solvent from solvent selection guides [21–24]. Our method is accurate, does not require additional instrumentation for species separation, avoids potentially harmful non-polar solvents, avoids emulsion formation, and provides accurate results in less than 2 h with less than 20 mL of waste generated per extraction.

## Experimental

### Reagents and instrumentation

In all cases, the purity of reagents and solvents was ACS Grade or better. Acid purity was at least Trace Metal Grade. Hydrochloric acid (HCl), nitric acid (HNO_3_), and hydrogen peroxide (H_2_O_2_) were purchased from Thermo Fisher Scientific (Waltham, MA, USA). Ethyl acetate, acetonitrile, sodium chloride (NaCl), sodium sulfate (Na_2_SO_4_), sodium thiosulfate (Na_2_S_2_O_3_), magnesium sulfate (MgSO_4_), l-cysteine hydrochloride monohydrate (l-cysteine•HCl•H_2_O), and l-cysteine were purchased from Thermo Fisher Scientific (Waltham, MA, USA) or MiliporeSigma (Rockville, MD, USA). Methylmercury standards at a nominal 2 ng/g concentration were purchased from BrooksRand Instruments (Seattle, WA, USA). Total mercury standards were purchased from Inorganic Ventures (Christiansburg, VA, USA). The absence of methylmercury in the Inorganic Ventures total mercury standards was confirmed with HPLC-ICP-MS.

Mercury and methylmercury were detected as total mercury using a Milestone DMA-80 evo (Sorisole, BG, Italy). Extracts were shaken with a Glas-Col shaker (Terre Haute, IN, USA) and centrifuged with a Sorvall X4R Pro centrifuge from Thermo Fisher Scientific (Waltham, MA, USA). For total mercury method comparison, samples were digested with a CEM MARS 6 microwave-assisted digestion system (CEM Corporation, Matthews, NC, USA). Digests were analyzed using an Agilent 8900 ICP-MS/MS (Agilent Technologies, Santa Clara, CA, USA). For methylmercury method comparison, samples were extracted on a DigiPREP MS 48-position hot block from SCP Science (Baie-D’Urfé, Quebec, Canada). Mercury species were separated using an Agilent 1200 HPLC (Agilent Technologies, Santa Clara, CA, USA) and detected by an Agilent 7900 ICP-MS. Separation was achieved on a Synergi Hydro-RP, 4 µm particle size, 150 × 4.6 mm C-18 chromatographic column (Phenomenex, Torrence, CA, USA).

### Mercury and methylmercury safety

Where possible, only trace (< 2 ng/g) methylmercury standards and extracts were handled. All total mercury and methylmercury standards and methylmercury extracts were prepared and handled in an exhausting fume hood. Analysts following the method described in the paper should follow their lab specific safety procedures when handling mercury and mercuric species as all forms of mercury are toxic and potentially dangerous to handle.

### Samples: acquisition and preparation

Except for two canned tuna samples, the samples used for validation were taken from a survey of the top 10 most consumed seafood species in the USA for per- and poly-fluoroalkyl substances [30]. All samples, including the two additional canned tuna samples, were purchased from online retailers or stores in the Washington, DC, Metropolitan area [30]. Samples were drained, if necessary, then homogenized and stored in IKA grinding mills (IKA Works, Inc., Wilmington, NC, USA) at − 30 °C. Samples were thawed at room temperature prior to analysis.

### Method comparison

Seventy-two samples were analyzed for total mercury by ICP-MS and TDA-AAS (Table S1). ICP-MS analyses followed Elemental Analysis Manual (EAM) 4.7 [5, 31]. Briefly, 8 mL HNO_3_ and 1 mL H_2_O_2_ were added to approximately 0.5 g of homogenized sample. Microwave-assisted digestion was carried out with a 25-min ramp to 200 °C and a 15-min hold. Digests were gravimetrically diluted to approximately 100 mL with water and 5 mL of 10% v/v HCl for a final acid concentration of 5% (v/v) HNO_3_ and 0.5% (v/v) HCl. Digests were analyzed for total mercury by monitoring the signal at mass-to-charge ratios 201 and 202. Quality control included method blanks, reference materials, and fortifications (i.e., spikes).

Two canned tuna samples were analyzed for methylmercury by HPLC-ICP-MS following EAM 4.8 [11, 32]. Briefly, 50 mL of an aqueous solution of 1% (w/v) l-cysteine•HCl•H_2_O was added to approximately 0.5 g of homogenized sample for 120 min at 60 °C on a hot block. Extracts were filtered through a 45-µm polyvinylidene fluoride filter. Methylmercury was separated from inorganic mercury with a C-18 column and an aqueous 0.1% (w/v) l-cysteine•HCl•H_2_O and 0.1% (w/v) l-cysteine isocratic mobile phase at a flow rate of 1 mL/min. The inorganic mercury and methylmercury species were quantified by monitoring the time-resolved signal at mass-to-charge ratios 201 and 202. Total mercury was estimated by summing the mass fractions for inorganic mercury and methylmercury. Quality control included method blanks, reference materials, and fortifications (i.e., spikes). All dilutions were performed gravimetrically.

### Mercury detection

**Fig. 1:**
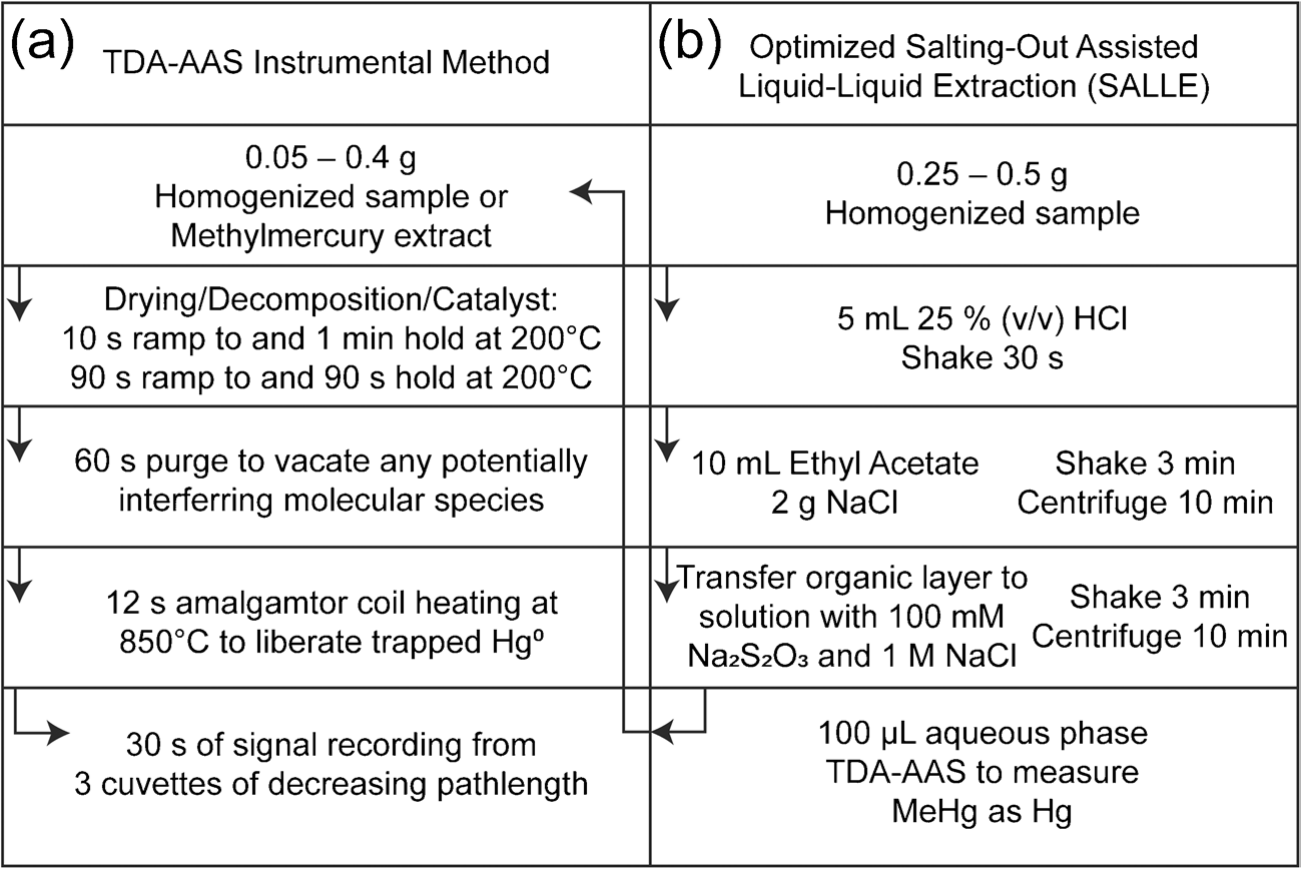
Schematic of the (**a**) thermal decomposition gold amalgamation atomic absorption spectrophotometry (TDA-AAS) instrumental method and (**b**) optimized salting-out assisted liquid–liquid extraction (SALLE) procedure

Total mercury measurements by TDA-AAS were carried out following a procedure adapted from the US Environmental Protection Agency (EPA) (Fig. 1a) [33]. Unless otherwise stated, 0.05–0.4 g of sample or methylmercury extract (see below) was introduced into an oxygen-rich combustion cell where the sample was dried then thermally decomposed in a temperature-controlled environment. All mercury species were reduced to Hg° in a catalyst section. Mercury was selectively trapped onto a gold amalgamator while potentially interfering molecular and halide species were removed by the carrier gas. After heating the amalgamator furnace, trapped mercury was freed from the amalgamator and directed into the spectrometer cells. Atomic absorption was carried out with a double beam spectrophotometer where mercury was quantitatively determined from absorbance at 253.65 nm [10]. The instrument was calibrated to cover the range 0–1000 ng.

### TDA-AAS calibration

Calibration was performed with reference materials or standard solutions diluted in 3% (v/v) HCl or 2 mM Na_2_S_2_O_3_ (Table 1). All liquid standards were loaded onto quartz sample boats, and all reference materials were loaded onto Ni alloy sample boats. Initial results with liquid standards showed more than 30% bias with ICP-MS measurements at concentrations > 100 ng/g. This was presumably due to instability of these solutions on the autosampler. Subsequent calibrations were conducted by loading four or fewer standards at a time to build the calibration curve. These calibrations with liquid standards led to results that were in better agreement with the ICP-MS measurements. Calibration curves from liquid standards were used for analyses of all methylmercury extracts.

**Table 1:** Reference materials used for instrument calibration. Certificate and uncertainty values listed for total mercury

Reference material	Certified value ± 95% uncertainty (ng/g)
NIST SRM 1568b Rice Flour	5.91 ± 0.36
NIST SRM 1570a Trace Elements in Spinach Leaves	29.7 ± 2.1
NIST SRM 1515 Apple Leaves	43.2 ± 2.3
NIST SRM 2976 Trace Elements and Methylmercury in Mussel Tissue (Freeze-Dried)	61.0 ± 3.6
NRC MESS-3 Marine Sediment	91 ± 9
NRC TORT-3 Lobster Hepatopancreas	292 ± 22
NRC DORM-4 Fish Protein	412 ± 36
NIST RM-50 Albacore Tuna	950 ± 100
NRC DOLT-4 Dogfish Liver	2580 ± 220

TDA-AAS is stable and does not require daily calibration [33]. Each instrumental run throughout method development and validation included at least one calibration check. These checks included analyzing a reference material or a standard prepared from a different mercury source than what was used for the instrument calibration, usually both. The instrument was recalibrated when two or more consecutive measurements of a calibration check resulted in a recovery outside the 80–115% and 90–110% ranges for checks at the 1 and 10 ng levels, respectively. These ranges were selected based on historic data collected throughout method development and validation.

### Methylmercury extraction: solvent selection

Various SALLE approaches to extracting methylmercury were evaluated to explore non-toluene-based extractions. Initial method development compared acetonitrile to ethyl acetate while using NaCl and MgSO_4_ or Na_2_SO_4_ to determine the optimal solvent. In general, 10 mL of 5 M HCl was added to approximately 0.5 g of sample and vigorously shaken for at least 30 s for initial liberation of mercury species. Next, 10 mL of either acetonitrile or ethyl acetate, 1 g of NaCl, and 4 g of MgSO_4_ or Na_2_SO_4_ were added. The sample was then shaken and centrifuged at 2000 g for 5 min or until distinct phases were observed. A portion of the organic phase was added to a 2 mM Na_2_S_2_O_3_ solution with or without 1 g of NaCl and 4 g of MgSO_4_ or Na_2_SO_4_. This was shaken then rested until two distinct phases were observed (approximately 10 min). A 100 µL portion of the aqueous phase was analyzed by TDA-AAS where methylmercury was quantified as mercury. All dilutions and solution transfers were performed gravimetrically.

### Methylmercury extraction: salt selection

We evaluated approximately equimolar concentrations of NaCl and MgSO_4_ in each of the two liquid–liquid extractions with ethyl acetate to determine the optimal salt configuration: (1) 10 mL of 3 M HCl and 10 mL of ethyl acetate, and (2) approximately 3 mL of ethyl acetate and 1 mL of an aqueous solution of either 2 mM or 100 mM Na_2_S_2_O_3_. Either 2 g of NaCl or 4 g of MgSO_4_ was added in the first extraction. Either an aqueous solution of Na_2_S_2_O_3_ and 1 M NaCl or Na_2_S_2_O_3_ and 1 M MgSO_4_ was used in the second extraction. Accuracy was determined from measuring methylmercury in IAEA 436a Tuna Fish Flesh Homogenate, and selectivity was evaluated from extractions of NIST SRM 2709 San Joaquin Soil and single element standards of mercury. The mercury in NIST SRM 2709 and the single element standards was 100% inorganic [34]. Therefore, any measurable signal from the instrument after extraction signified the extent of potential false positive bias.

### Methylmercury stability evaluation

The stability of 100 ng/g solutions of methylmercury prepared in 0.5% (v/v) acetic acid and 0.2% (v/v) HCl, 10 mM Se and 1 M NaCl, 10 mM Na_2_S_2_O_3_, 10 mM Na_2_S_2_O_3_ + 1 M NaCl + 10 mM Se, 100 mM l-cysteine·HCl·H_2_O and 1 M NaCl, 100 mM Na_2_S_2_O_3_ and 1 M NaCl, and 3% (v/v) HCl on the TDS-AAS autosampler was evaluated to determine the stability of diluted methylmercury standard solutions in varied aqueous matrices. The stability of methylmercury sample extracts on the autosampler was first evaluated by repeated measurements of IAEA 436a Tuna Fish Flesh Homogenate. The autosampler is a part of the instrument and is not temperature controlled. Extracts sit at room temperature until analysis. Additional stability tests were carried out during validation. Duplicate instrumental measurements of extracts were performed at various points of the autosampler sequence. These extracts were acquired from using the optimized extraction procedure described below. Typically, the first instrumental replicate was measured for all extracts, then at the end of the sequence, one or more extracts spanning the sequence were analyzed again. The relative percent difference between the repeated and initial instrumental replicate was calculated to signify the degree of stability of the extracts on the autosampler. All extracts were delivered and massed into quartz vessel boats. Because each instrumental analysis consumed the matrix, all instrumental replicates represent distinct additions of standard or extract into a vessel boat.

The daily stability of IAEA 476 Fish Homogenate and NIST RM-50 Albacore Tuna methylmercury extracts in polypropylene plastic and amber glass was evaluated for 6 days, with measurements taken at 0, 1, 4, and 6 days after extraction. The optimized procedure in “Methylmercury extraction: optimized procedure” was followed, except a 10 mM Na_2_S_2_O_3_ and 1 M NaCl aqueous solution were used in the second extraction with ethyl acetate. From this extraction, a 0.5 mL aliquot was delivered and kept in an amber glass HPLC vial. The remaining 0.5 mL was kept in the original polypropylene 15-mL conical tube. All extracts were kept at 4 °C between daily measurements.

### Methylmercury extraction: optimized procedure

Our approach derived from the procedure developed by Azemard and Vassileva [19]. We substituted ethyl acetate in place of toluene and optimized the amount of reagents used for extraction based on analytical recovery and the amount of waste generated. The optimized procedure featured two extractions (Fig. 1b). First, 5 mL of 3 M (25% v/v) HCl was mixed with a 0.5 g portion of sample in a 50-mL conical polypropylene tube to liberate methylmercury and inorganic mercury from the matrix by forming non-polar and polar methylmercury chloride and inorganic mercury chloride. Then, 10 mL of ethyl acetate and 2 g of NaCl were added to the same tube to separate methylmercury chloride from inorganic mercury chloride. After shaking for 30 s and centrifugation for 10 min at 3800 g, a portion of the organic phase was added to a 15-mL conical polypropylene tube with 1 mL of a 1 M NaCl and 100 mM Na_2_S_2_O_3_ solution. Here, mercury from methylmercury chloride was covalently bound to sulfur from the thiosulfate ion and stripped away from the organic phase. The sample was mixed via shaking for 30 s and centrifuged for 10 min at 3800 g; then 100 µL (~ 0.1 g) of the aqueous phase following centrifugation was analyzed by TDA-AAS where methylmercury was quantified as mercury. All dilutions and solution transfers were performed gravimetrically.

### Methylmercury method limit of detection and quantification

The limit of detection (LOD) and limit of quantification (LOQ) of the methylmercury method was determined with NIST 1566b oyster tissue according to Eqs. 1 and 2 from FDA’s Elemental Analysis Manual guidelines [35], where *s* is the standard deviation of NIST 1566b measurements, ${t}_{95}$ is the one-sided Student’s *t* at 95% confidence level, and *n* is the number of NIST 1566b measurements.

1 $$\text{LOD}=2 \times {t}_{95}\times s\times \sqrt{1+\frac{1}{n}}$$

2 $$\text{LOQ}=30s$$

### Methylmercury method validation: reference materials

Seven reference materials with varying matrices, mercury concentrations, and fractions of methylmercury to total mercury were evaluated three or more times for validation (Table S2). Some expired reference materials (e.g., NRC DOLT-3, NRC DORM-3) were included due to the limited number of available reference materials with certified values for methylmercury. NIST RM-50 Albacore Tuna lacks a certified value for methylmercury; however, several papers have reported values for methylmercury in this material. We used the average and standard deviation of the reported values for validation [11, 36–38].

*Z* scores were determined according to Eq. 3 [39], where ${X}_{\text{meas}}$ is the measurement result, ${X}_{\text{ref}}$ is the reference value found on the certificate of analysis, and $\sigma$ = (*σ*_meas_^2^ + *σ*_ref_^2^)^1/2^, where *σ*_meas_ is the uncertainty of the measurement and *σ*_ref_ is the uncertainty of the reference value. Reference material measurement results were compared with certified values given on the certificates.

3 $$Z=({X}_{\text{meas}}-{X}_{\text{ref}})/\sigma$$

The total standard uncertainty for reference material certified values (*σ*_ref_) was obtained from the certificate. Because the uncertainties were listed as expanded uncertainties at the 95% confidence level, they were divided by the coverage factor listed on the certificate (e.g., *k* = 2) to obtain standard uncertainties at approximately a 67% confidence level for use in *Z* score calculations. Total standard uncertainty for measurement results (*σ*_meas_) was defined at 10% when greater than the LOQ.

### Methylmercury method validation: fortified samples

Samples of cod (Cod 1–8), crab (Crab 1–8), pollock (Pollock 1–5), and tuna (Tuna 1–8, Tuna 035, Tuna 036) were used for addition recovery experiments (Table S1). Samples were fortified with methylmercury standards or reference materials in triplicate at 50, 100, and 200% of the native concentration. The native concentration was determined prior to fortification experiments from one or more analytical portions using the optimized procedure (Fig. 1b). Methylmercury standards were diluted in 2–3% (v/v) HCl. NIST RM-50 Albacore Tuna, IAEA 476 Fish Homogenate, and NIST 2976 Mussel Tissue were used for fortification with reference materials depending on the native concentration of the sample. Samples were originally fortified with methylmercury standards. Fortification with reference materials was carried out after observing variability in fortification results using methylmercury standards.

### Total Mercury Method Validation: Reference Materials and Fortified Analytical Portions

Seven reference materials (Table S3) with certified mercury concentrations ranging from 37.1 to 4260 ng/g were used for validation. Each reference material was analyzed six or more times spanning 2 or more days. Samples of clam (Clam 1–8), crab (Crab 1–8), shrimp (Shrimp 1–3), and tuna (Tuna 1–5) were used for addition recovery experiments (Table S1). Samples were fortified in duplicate at 50, 100, and 200% of the native concentration. Mercury standards used for fortification were diluted in 3% (v/v) HCl. *Z* scores and the LOD and LOQ were determined according to what was described above in “Methylmercury method limit of detection and quantification.” NIST 1568b was analyzed 30 times, and the standard deviation from these results was used to determine the LOD and LOQ for total mercury.

## Results and discussion

### Methylmercury method development: solvent selection

Several acidic halide- and toluene-based methylmercury extractions have led to deleterious emulsion formation causing variation in analytical recovery [12, 17, 40]. Our initial method development with NRC DOLT-3 and NRC DOLT-4 reference materials confirmed these findings. Applying the method developed by Barghigiani and Scerbo on DOLT-4 led to an average recovery of 99 ± 12% (*n* = 3, 2 σ). This method required two additions of toluene. To reduce waste, we evaluated whether a single toluene extraction would work. DOLT-3 was analyzed with and without a second toluene addition. This led to average recoveries of 94 ± 8 and 70 ± 4% (*n* = 3, 2 σ), respectively, suggesting two additions of toluene were needed for accurate recovery, confirming what was reported by Maggi et al. [17]. However, an emulsion formed at the HBr-toluene interface for both analyses of DOLT-3, regardless of the number of toluene additions. This is consistent with what was reported by Barghigiani and Scerbo and Maggi et al. [12, 17]. Additional centrifugation and 1 mL additions of isopropanol were used to break the emulsion. These results suggest methods that are capable of phase separation without emulsion formation are necessary for consistently accurate results.

Initial method development compared acetonitrile to ethyl acetate. When adding 1 g of NaCl and 4 g of MgSO_4_ or Na_2_SO_4_ to both the initial extraction with HCl and organic solvent, and the second extraction of organic solvent with an aqueous solution of Na_2_S_2_O_3_, the average recovery from DOLT-3 was 130 ± 18 and 95 ± 12% (*n* = 3, 2 σ) with acetonitrile and ethyl acetate, respectively. The varied and high recovery of methylmercury in acetonitrile may be due to co-extraction of inorganic mercury from the water-rich phase in the first extraction where residual water may be present in the acetonitrile phase even at salt saturation [28]. These results suggest ethyl acetate is a suitable replacement for toluene for SALLE extractions of methylmercury. In addition to its analytical utility, ethyl acetate is a less hazardous solvent with less impact on the environment when compared to acetonitrile and toluene [21–24].

### Methylmercury method development: salt selection

At low pH in a Cl environment, methylmercury and inorganic mercury readily form and predominantly exist as the non-polar and polar species, CH_3_HgCl and HgCl_4_^2−^, respectively [41, 42], and methylmercury and inorganic mercury form stable bonds with thiol ligands [4, 43, 44]. Initial liberation of mercury from the sample matrix with dilute HCl allows for the formation of the polar and non-polar species of inorganic mercury and methylmercury [41, 45, 46]. Once these species are formed, separation of methylmercury follows from classical liquid–liquid extraction, where methylmercury partitions into the non-polar phase. Adding an aliquot of the organic phase to an aqueous solution of an S-containing molecule (e.g., l-cysteine, Na_2_S_2_O_3_) allows for the formation of methylmercury-SH bound molecules that partition into the aqueous phase [46]. We evaluated the effect of salt type and concentration on the extraction recovery of methylmercury from the double liquid–liquid extraction.

Ion solvent interactions are characterized by the Debye-Hückel theory, which mathematically describes the non-ideality of electrolyte solutions [47]. However, at the elevated salt concentrations (e.g., > 0.1 M) routinely used in analytical extractions, ion-specific effects dominate, and Debye-Hückel theory does not match empirical measurements [47, 48]. In these cases, the degree of salting-out is governed by the anion of the salt, where SO_4_^2−^ and Cl⁻ have both been shown to favorably salt-out organic solvents from water [47].

Salt in a SALLE extraction with acetonitrile affects phase separation and the extraction of analytes in each phase by affecting physical and chemical properties such as hydration, Born energy, and electrostatic interaction [28]. Salts that form complexes in partially miscible solutions affect the migration and convection rate of droplets where liquid–liquid phase separations of partially miscible solutions occur as a function of droplet growth, diffusion coalescence, and convection coalescence [29]. Two detailed studies of salts in water-acetonitrile-salt and water-2-butoxyethanol-salt systems identified NaCl and MgSO_4_ as effective for phase partitioning and analyte separation across the two phases [28, 29]. This agrees with more empirical studies which highlighted the popularity of NaCl and MgSO_4_ in SALLE extractions [25].

**Fig. 2:**
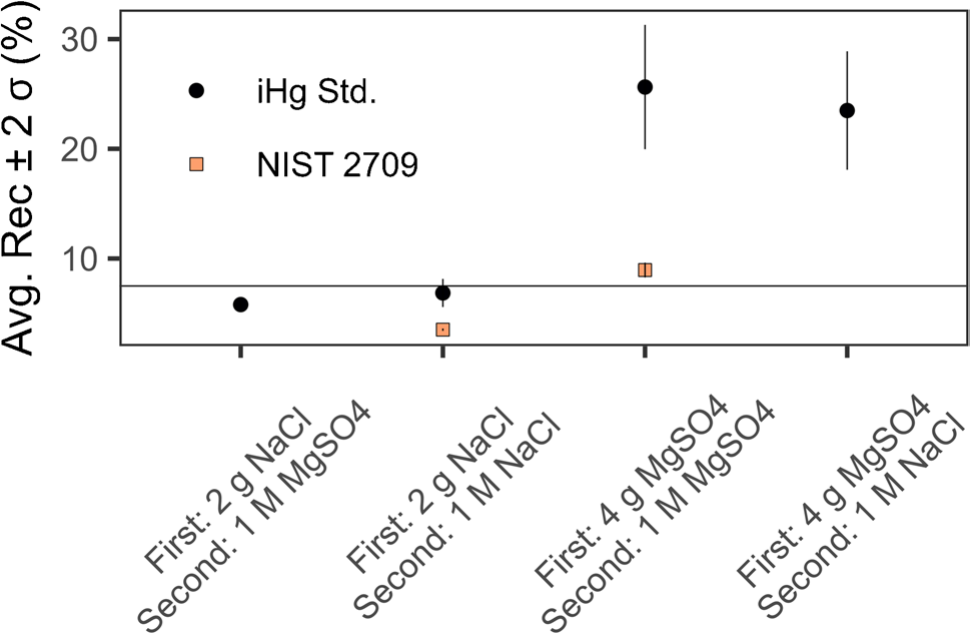
Selectivity results from analyzing inorganic mercury standards or reference materials. The amount and type of salt in the first and second extraction are listed on the *x*-axis for clarity. The horizontal line represents a 7.5% false positive bias

Using either NaCl or MgSO_4_ led to accurate results for measurements of methylmercury in IAEA 436a with average recoveries of 99 ± 11% (*n* = 9, 2σ) and 94 ± 13% (*n* = 20, 2σ), respectively. The selectivity, however, varied significantly according to what salt was used in the first extraction (Fig. 2). The mercury in NIST SRM 2709 and the single element standards was 100% inorganic [34]. Therefore, any measurable signal from the instrument after extraction signified the extent of potential false positive bias. The difference in results for what salt was used in the second extraction with ethyl acetate and the aqueous solution of Na_2_S_2_O_3_ was much lower. In finfish with elevated levels of mercury, > 90% of the mercury present is methylmercury [11, 49]. As a result, the potential for false positive bias when using NaCl is insignificant. The developed SALLE method extracted at most 7.5% of the inorganic mercury from the tested standards and reference material with 100% Hg present in the inorganic form. In a hypothetical finfish with 90% mercury present as methylmercury, if we assume a worst case scenario where 7.5% of the inorganic mercury is coextracted, then there would be a < 1% false positive error for methylmercury. These results suggest NaCl is a suitable salt that is accurate and selective for SALLE isolation of methylmercury from finfish.

There are different types of salts and salt concentrations reported in published methylmercury extraction methods for TDA-AAS detection. For example, Kim et al. used 25% NaCl in the initial extraction and separation of methylmercury from inorganic mercury, while Scerbo and Barghigiani did not use any salt [12, 20]. Scerbo and Barghigiani used Na_2_SO_4_ and sodium acetate with l-cysteine in their method’s second extraction, where mercury from methylmercury was bound to sulfur from cysteine, while Vassileva and Azemard did not use any additional salt with their sulfur source, Na_2_S_2_O_3_. We show NaCl and Na_2_S_2_O_3_ are capable of accurate and efficient methylmercury extractions from finfish from two liquid–liquid extractions with ethyl acetate.

### Methylmercury method development: methylmercury stability

The lack of stability of mercury species is well-known. Factors such as the concentration of mercury, vessel material, complexing agent, pH, and light have been shown to be causes of instability, where mercury is lost due to adsorption on the container wall, volatilization, and species conversion between elemental, inorganic, and organic mercury [11, 50, 51]. Considering methylmercury extraction for TDA-AAS detection, Azemard and Vassileva reported extract stability at room temperature for up to 5 h and daily stability for up to 2 days with storage at 4 °C [19]. White et al., however, reported almost immediate loss of methylmercury from 100 ng/L methylmercury chloride and methylmercury hydroxide solutions on their instrument autosampler [52].

**Fig. 3:**
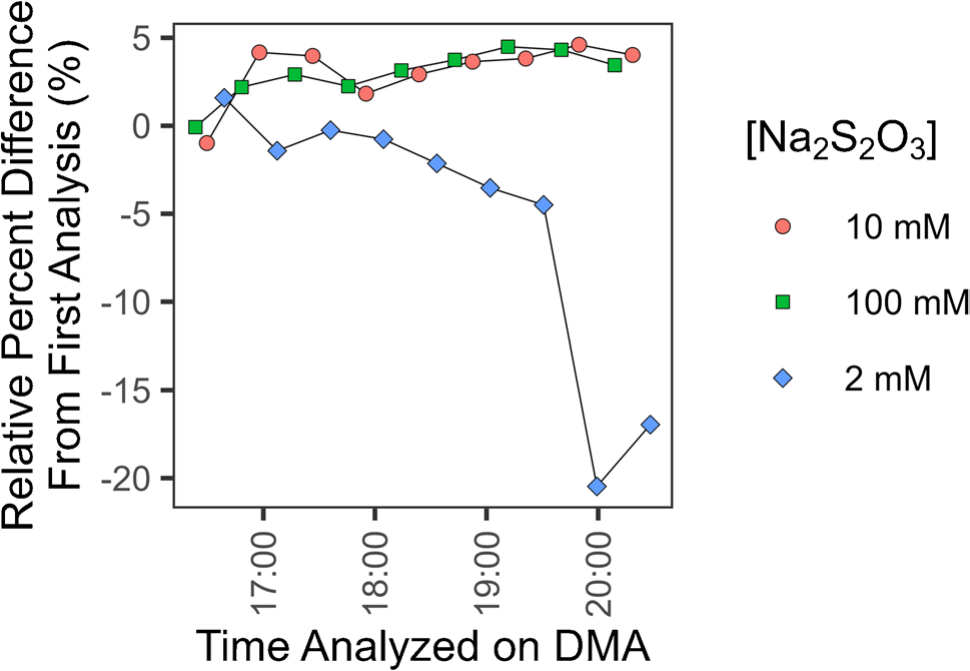
Stability results from IAEA 436a extracts on the instrument autosampler. All extracts prepared in 1 M NaCl. The concentration of Na_2_S_2_O_3_ in the extracts varied from 2 to 10 mM

**Fig. 4:**
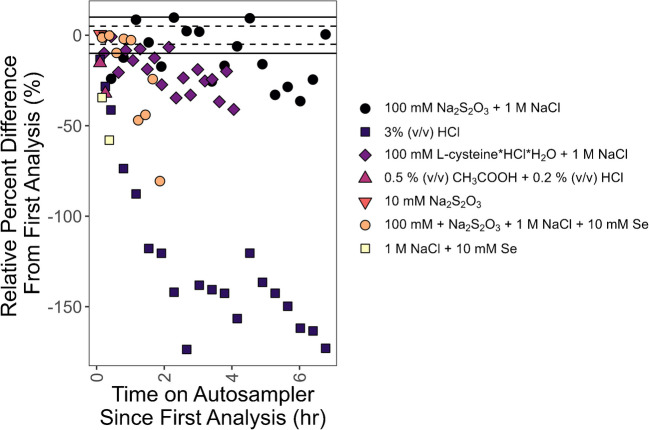
Stability results on the instrument autosampler from analyzing methylmercury standards prepared in varied aqueous solutions

IAEA 436a methylmercury extracts were stable on the autosampler for up to 4 h in aqueous solutions of 1 M NaCl and 10 mM or 100 mM Na_2_S_2_O_3_. IAEA 436a extracts in aqueous solutions of 1 M NaCl and 2 mM Na_2_S_2_O_3_, however, were only stable for up to 2 h (Fig. 3). There was significant instability for methylmercury standards prepared in various aqueous solutions (Fig. 4). All 100 ng/g aqueous solutions of methylmercury demonstrated more than 10% loss after 4 h of measurement. Solutions prepared in 100 mM Na_2_S_2_O_3_ and 1 M NaCl, and 100 mM l-cysteine·HCl·H_2_O and 1 M NaCl, were the most stable with decreasing trends and average recoveries of 86 ± 23 and 88 ± 18% (2σ), respectively, for up to 4 h on the autosampler. The solutions prepared in 3% (v/v) HCl, 10 mM selenium, and 0.5% (v/v) acetic acid and 0.2% (v/v) HCl were not stable and showed immediate loss. The addition of sulfur from a thiol-containing molecule (e.g., Na_2_S_2_O_3_ and l-cysteine) increased the stability of mercury in the solution agreeing with what was shown in White et al. who used a thiol-containing absorbent in the autosampler vessel sample boat [52].

**Fig. 5:**
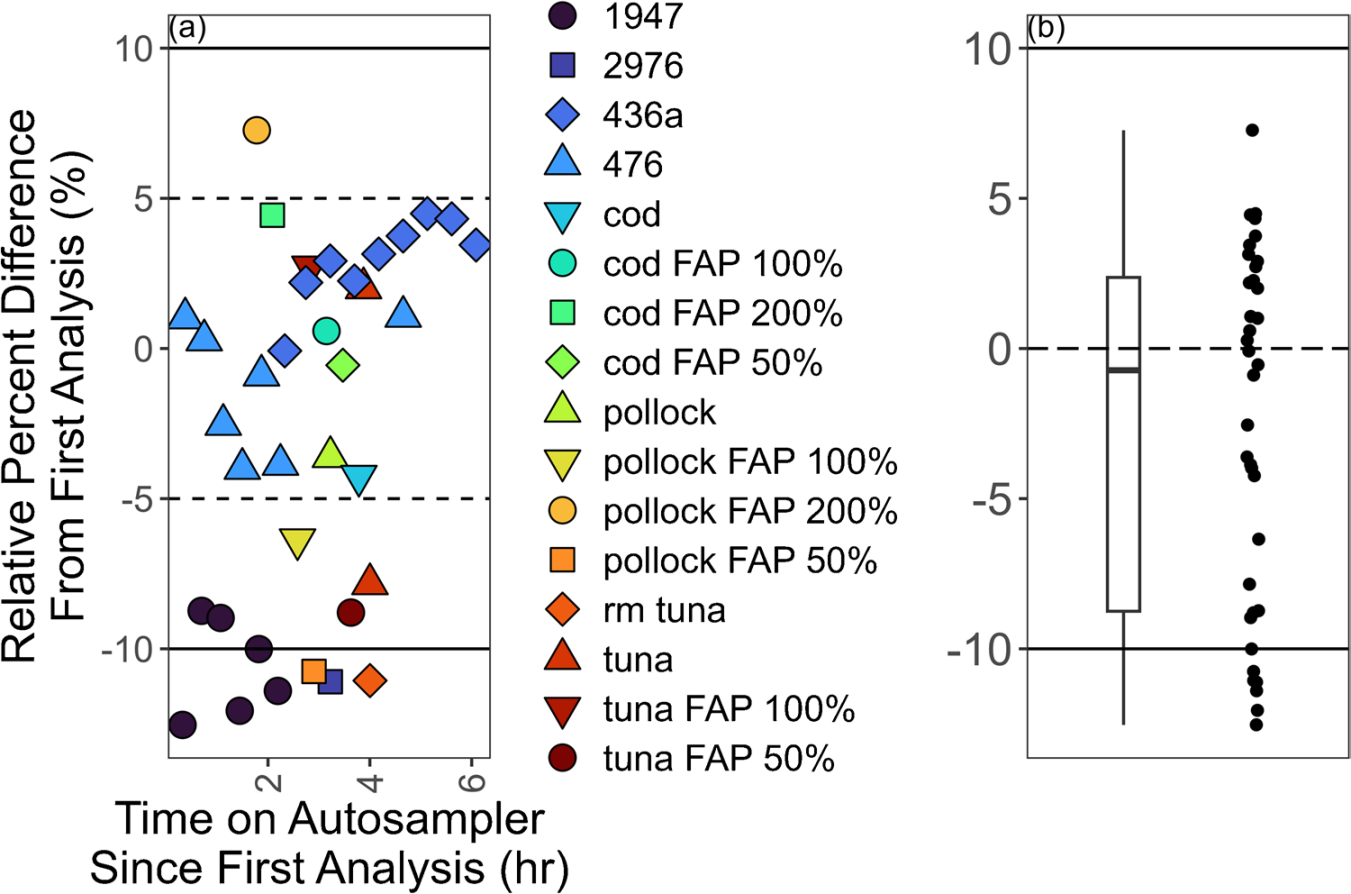
Stability results on the instrument autosampler from analyzing methylmercury extracts. (**a**) Results displayed as a function of time between the initial and the repeated analysis. The dashed and solid lines represent the ranges corresponding to ± 5 and 10% relative percent difference (RPD) from the first analysis. (**b**) Results summarized as a boxplot and point overlay. The dashed line marks 0% RPD from the first analysis, and the solid lines represent the ± 10% RPD range

Methylmercury extracts from samples were more stable than standards for all sample matrices and concentrations evaluated (Fig. 5). This suggests a coextracted component(s) from the matrix aids in the stability of methylmercury in solution. Mercury’s affinity to selenium is well-known and has been proposed to play a role in an antagonistic pathway for toxicity where mercury binds to available selenium and forms insoluble HgSe particles that are excreted by potentially contaminated organisms (e.g., pilot whales and seabirds) [53, 54]. Adding selenium at 10 mM concentrations to the standard solutions, however, did not stabilize methylmercury, suggesting another factor is at play in the stability of SALLE extracts (Fig. 5). Identifying this additional factor or factors, however, was outside the scope of this study. From these results, we selected an aqueous solution with concentrations of 1 M NaCl and 100 mM Na_2_S_2_O_3_ for the second extraction with ethyl acetate.

**Fig. 6:**
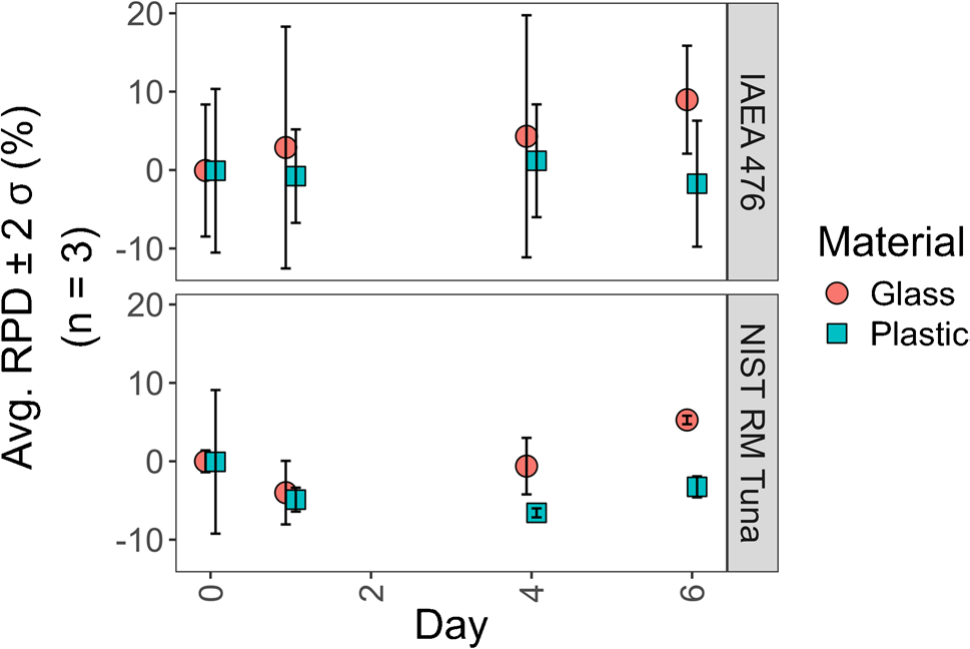
Stability results from analyzing IAEA 476 and NIST RM-50 Albacore Tuna methylmercury extracts stored in glass or polypropylene plastic. The relative percent difference (RPD) from the first analysis (i.e., day 0) was determined for each replicate. The average of the day 0 concentrations was considered to determine the RPD for each replicate across the different analysis days

Extracts stored in amber glass and polyethylene tubes at 4 °C were stable for 24 h (Fig. 6). Except for the initial measurements of NIST RM-50 Albacore Tuna and IAEA 476 Fish homogenate, all concentrations from samples stored in amber glass containers were higher than those stored in plastic tubes. All measurements from extracts stored in glass and plastic tubes for IAEA 476 were within two standard deviations of each other, while the variation in the replicates was slightly larger from extracts stored in glass. The fourth- and sixth-day measurements of NIST RM-50, however, showed differences greater than two standard deviations between extracts stored in polyethylene and glass containers. From these results, we recommend immediate analysis of extracts after SALLE. If an immediate analysis cannot be done, storing the extract in the original container at 4 °C and analyzing within the working day is allowed. We recommend storing the extracts in amber glass vials at 4 °C if storing overnight and discarding any extracts stored for more than 24 h. Our results agree with previous findings while providing more detail on how to ensure stability for TDA-AAS measurements [11, 50–52].

### Methylmercury method validation: reference materials

**Fig. 7:**
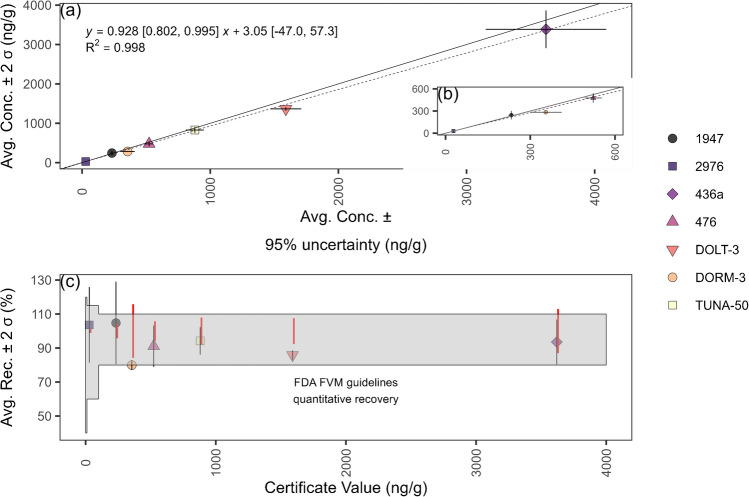
Results from analyzing methylmercury in reference materials. (**a**) Observed concentrations from the optimized methylmercury method compared to reference material certified values. The solid and dashed lines represent a line with a slope of 1 (i.e., perfect agreement with the reference material) and the fitted line between observed and theoretical concentrations using Passing-Bablok regression, respectively [64]. The brackets show the 95% confidence interval for the slope and intercept. (**b**) Results from (**a**) zoomed in on the range 0–600 ng/g. (**c**) Recoveries compared to reference material uncertainty. The solid vertical line is the 95% uncertainty range for the reference material. The shaded region represents the quantitative recovery range from the FDA chemical methods validation guidelines [55]

Excluding NRC TORT-3 and considering the average of three or more replicates, recoveries from analyzing reference materials for methylmercury ranged from 80 to 105%. The overall average recovery considering each individual replicate was 95 ± 18% (*n* = 82, 2σ) (Figs. 7 and S1a, Table S2). The average recovery for NRC TORT-3, however, was 157 ± 54% (*n* = 5, 2 σ). Excluding NRC TORT-3, *Z* scores considering individual replicates ranged from − 1.98 to 1.65 with a median of − 0.56 (Fig. S1b). Analyzing NRC TORT-3 by HPLC-ICP-MS led to an average recovery of 81 ± 12% (*n* = 6, 2σ). Except for TORT-3, all results were within the FDA chemical methods validation guidelines quantitative recovery range (Fig. 7c) [55]. The determined LOD and LOQ from analyzing NIST SRM 1566b 7 times were 3.8 and 27 ng/g, respectively.

TORT-3 is a lobster hepatopancreas material. The varied and high recovery from applying the developed methylmercury extraction procedure on this material was not evaluated further because finfish was the targeted sample matrix. Possible reasons for inefficiencies in extraction may be due to how methylmercury is bound to proteins in lobster hepatopancreas. Another potential cause may be the relatively high lipid (e.g., 40%) content in hepatopancreas, affecting phase separation from the non-polar and polar extraction solutions [56]. Inaccuracies have been reported for toluene extractions of methylmercury from a dogfish liver reference material, another relatively high lipid content material [17], whereas accurate results were obtained when applying the developed SALLE ethyl acetate approach (Fig. 7). Interestingly, SALLE extractions with toluene did not lead to high recovery for TORT-3, suggesting ethyl acetate may not be suitable for methylmercury extractions from lobster, specifically lobster hepatopancreas. In addition to finfish, accurate results were observed for methylmercury extractions from mussel tissue (Table S2). However, application for matrices other than finfish requires careful optimization of the method.

Our results compare well to similar methods in the literature. From five reports, 22 different reference materials were analyzed with a methylmercury extraction and TDA-AAS detection method [12, 17–20]. The recoveries reported ranged from 74 to 144%, with an overall average recovery of 97 ± 18% (2σ). These methods all used toluene as the organic solvent and reported acceptable recoveries for TORT reference materials, confirming toluene may be needed for accurate results in this matrix.

### Methylmercury method matrix effect validation: fortified analytical portions

**Fig. 8:**
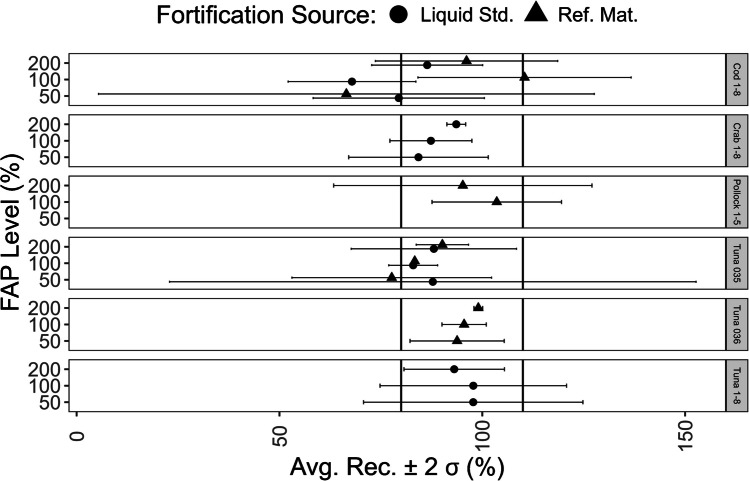
Methylmercury addition recovery results from fortified analytical portions (FAP)s

Considering the average recovery from triplicate preparations, recoveries from fortified analytical portions (FAP) ranged from 66 to 110%, with an average recovery of 89 ± 21% (*n* = 23, 2σ) (Fig. 8). Variability tended to decrease with increasing amounts of fortification except for the pollock fortifications (Fig. 8). The pollock sample had the lowest native concentration of methylmercury (20 ± 6 ng/g, *n* = 3, 2σ). The concentrations determined in the first level pollock FAP measurements were < LOQ and not considered. The variation in the pollock FAP results may be due to operating near the method LOQ.

Considering all replicates, 19 of the 23 were within the 80–110% recovery range. The four replicates outside the range were biased low. Presumably this was due to loss of methylmercury from the extract in addition to the loss of methylmercury from the fortification during the entire extraction and detection procedure. The variability in these results may be due to the instability of methylmercury in the solutions and the inhomogeneity of the reference materials used for fortification. These two factors compounded with the method uncertainty overall led to an average relative standard deviation of 11%, with a minimum and maximum relative standard deviation of 0.3 and 50%, respectively, for the triplicate fortifications.

### Methylmercury validation: comparison to HPLC-ICP-MS and other methods

Two canned tuna samples were analyzed by the developed method and EAM 4.8, a validated HPLC-ICP-MS method [11]. Results from the optimized method were within two standard deviations of the results from HPLC-ICP-MS (Table 2). In conjunction with the results from “Total mercury method validation: comparison to ICP-MS,” this suggests the developed non-chromatographic method is accurate and comparable to complementary ICP-MS-based techniques.

**Table 2:** Summarized results from analyzing two canned tuna samples using two different methods. The mean and two standard deviations are reported. Triplicate analytical portions were subjected to each method for each sample except for canned tuna 1 by SALLE-TDA-AAS. Nine analytical portions of canned tuna 1 were analyzed by SALLE-TDA-AAS

Sample	HPLC-ICP-MS	SALLE-TDA-AAS
Canned tuna 1	642 ± 10	601 ± 86
Canned tuna 2	606 ± 21	564 ± 24

Several techniques have been used and described in the literature to detect and measure methylmercury. Most detection strategies involve two components: (i) a separation component to isolate methylmercury, and (ii) a detection system. As a result, the instruments used to measure methylmercury range in complexity and cost as a function of the choice in separating and detecting the analyte. These include chemical speciation using HPLC, gas chromatography (GC), and anion exchange column chromatography with detection by ICP-MS, CV-AAS, and CV-AFS [13, 57–59]. Techniques to extract methylmercury include solid phase extraction, liquid–liquid extraction, liquid phase microextraction, cloud point extraction, and microwave-assisted extraction, where the choice of extraction strategy depends on concentration level and detection technique [13, 58]. ICP-MS and CV-AFS provide low detection limits with reported method detection limits of 0.030 and 0.042 pg, respectively, after GC separation [60].

Our approach does not achieve these low levels of detection as the target concentration of mercury in finfish is not as low as natural waters [11, 61]. However, the SALLE-TDA-AAS method provides comparable accuracy at a fraction of the cost and time required to speciate finfish samples, especially when compared to ICP-detection methods. We eliminate the need for an additional instrumental component as the analyte is selectively isolated from careful optimization of extraction conditions. Our choice of detection, TDA-AAS, allows for relatively cheap and facile analysis of mercury, complementing routine screening of samples for total mercury.

### Total mercury method validation: reference materials and fortified analytical portions

**Fig. 9:**
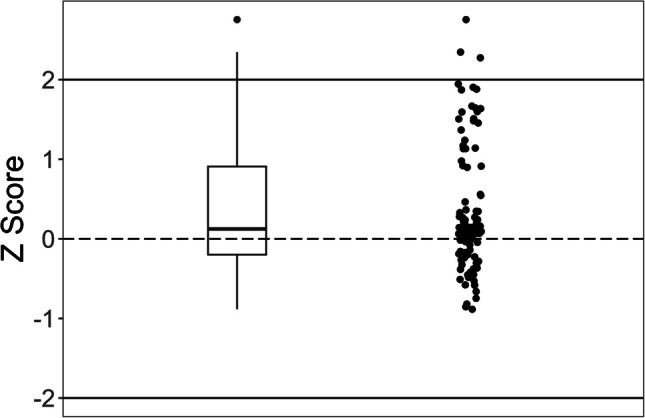
*Z* scores for total mercury from reference material analyses summarized as a boxplot and point overlay

Considering the average recovery from six or more replicates for each reference material, recoveries from analyzing reference materials for total mercury ranged from 96 to 118%. Considering each individual reference material replicate, the average recovery was 104 ± 24% (*n* = 102, 2σ), and *Z* scores ranged from − 0.88 to 2.75 with a median of 0.12 (Fig. 9, Table S3). The LOD and the LOQ for total mercury were 2.8 and 25 ng/g, respectively.

The agreement with certified values from several reference materials confirmed TDA-AAS is an accurate technique for total mercury determination. Of the 102 reference material replicates, two replicates of NIST RM-50 and one replicate of IAEA 436a had *Z* scores outside the − 2 to 2 range (Table S3). Presumably, these high recoveries of mercury were due to potential carry-over on the instrument from previous instrument analyses as a result of the elevated levels of mercury in these reference materials [9]. Even so, 97% of the results were in good agreement with reference material certified values and their uncertainty.

**Fig. 10:**
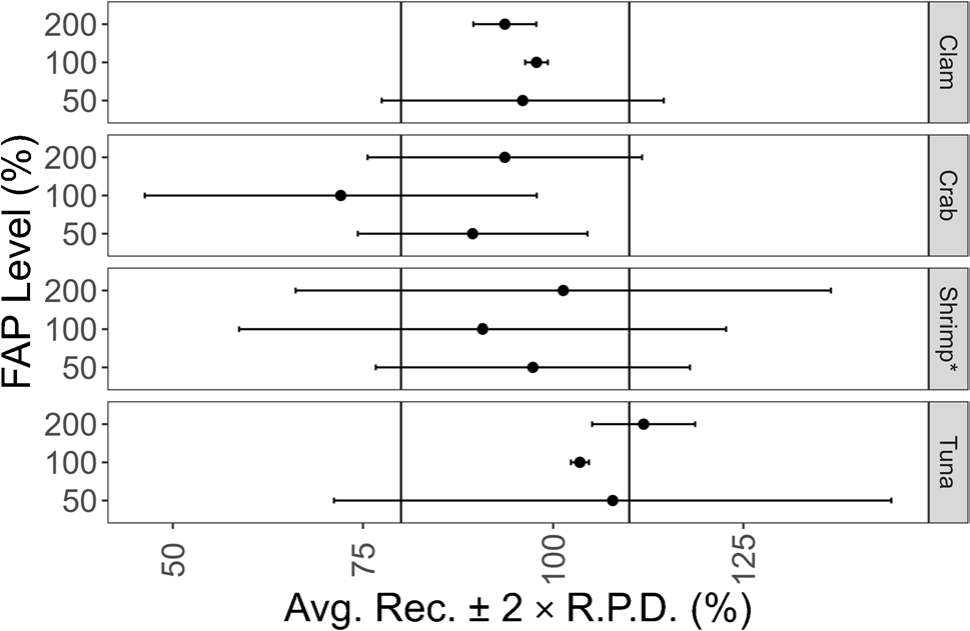
Total mercury addition recovery results from fortified analytical portions (FAP)s. All shrimp sample fortifications were prepared at or below method LOQ

Considering individual replicates, recoveries from analyzing FAPs ranged from 72 to 112%, with an average recovery of 96 ± 20% (*n* = 12, 2σ) (Fig. 10). Similar to methylmercury, the variation in total mercury FAP results may be due to the instability of mercury in the standard solution used for fortification and the instability of the fortification on the instrument autosampler in the autosampler boat. The average relative percent difference (RPD) from the duplicate fortifications was 9%, with a minimum and maximum of 0.6 and 18%, respectively. Inorganic mercury, the source of mercury used for all total mercury fortifications, has been shown to be stable on the instrument autosampler for over 4 h [52]. Our work agrees, considering results from analyzing calibration check solutions across the instrument sequence for multiple instrumental runs. The interaction of the fortification with the sample and the Ni alloy sample boat may have caused the instability. In general, liquid samples should be placed in and analyzed with quartz vessel sample boats according to manufacturer instructions. We used Ni alloy sample boats for all solid samples and fortified on top with liquid standards of mercury in 3% (v/v) HCl. The interaction of the fortification solution matrix with the alloy may have led to decreased stability.

All shrimp sample fortifications were prepared at or below method LOQ (e.g., 3–25 ng/g) to evaluate performance in the trace region of the method. All observed concentrations from shrimp FAPs were < LOQ except for one of the two replicates of the level two FAPs. Operating below the LOQ of the method contributed to the increased variation in results observed for the shrimp FAPs. The average recovery from duplicate analyses of an FAP was within the 80–110% range for 10 of the 12 FAPs analyzed, suggesting TDA-AAS was accurate for total mercury determination in matrices of finfish, clam, and shrimp (Fig. 10). Working with mercury in solution, from preparing fortification solutions to fortifying samples, with TDA-AAS instrumentation presents a unique challenge as all mercury species are volatile and interact with vessel material [50]. Caution should be taken to preserve mercury in the matrix prior to thermal degradation in the instrument when performing addition recovery experiments.

### Total mercury method validation: comparison to ICP-MS

**Fig. 11:**
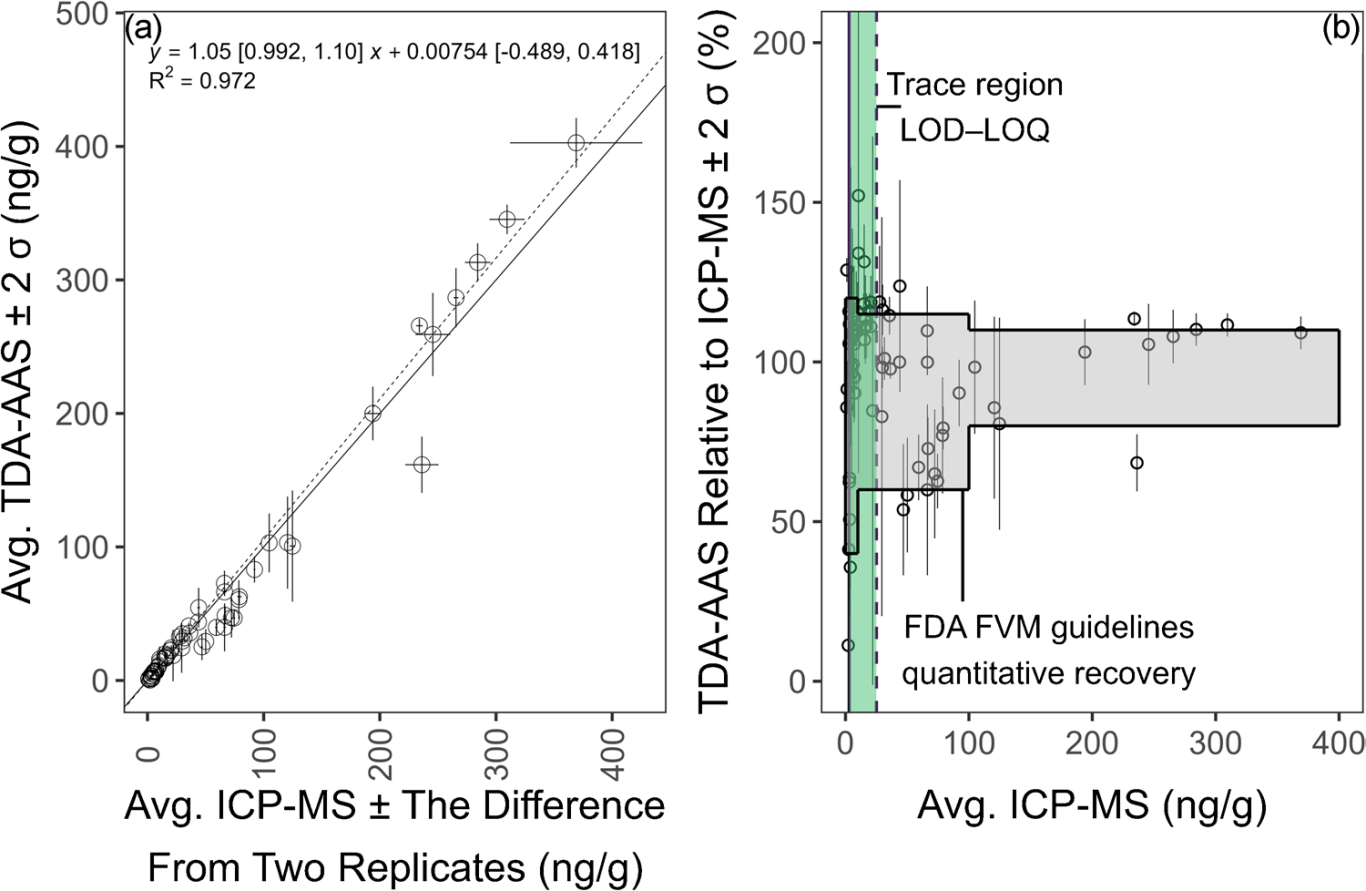
Thermal decomposition gold amalgamation atomic absorption spectrophotometry (TDA-AAS) total mercury results compared to results from ICP-MS analyses following EAM 4.7. (**a**) The solid and dashed lines represent a line with a slope of 1 (i.e., perfect agreement between the two techniques) and the fitted line between TDA-AAS and ICP-MS concentrations, respectively. The fitted line was computed using Passing-Bablok regression [64]. The brackets show the 95% confidence internal for the slope and intercept. The error bars for the *x*-axis represent the difference between two replicates from ICP-MS analysis using EAM 4.7. The error bars for the *y*-axis represent two standard deviations from analyzing three analytical portions using TDA-AAS. (**b**) TDA-AAS results relative to ICP-MS concentration as a percentage. The error bars for the *y*-axis represent two standard deviations from analyzing three portions using TDA-AAS. The shaded region represents the quantitative recovery range from the FDA chemical methods validation guidelines [55]. This assumed the concentrations from ICP-MS analyses were the theoretical values

Our results reaffirm TDA-AAS provides comparable results to ICP-MS (Fig. 11). Of the 77 samples evaluated by both techniques, 33 were > LOQ for TDA-AAS. According to FDA chemical methods validation guidelines, of the 33 samples with mercury concentrations > LOQ, 31 were within the quantitative recovery range at two standard deviations when considering the value obtained from ICP-MS as the theoretical concentration of mercury in the sample (Fig. 11) [55]. Furthermore, considering the fitted line using Passing-Bablok regression to take into account the uncertainty in the independent (i.e., ICP-MS results) and dependent variables (i.e., TDA-AAS results), the 95% confidence intervals for the slope and the intercept include 1 and 0, respectively, suggesting TDA-AAS and ICP-MS results are comparable. This agrees with a recent report by Neto et al. [62] who showed TDA-AAS measurements of mercury in Amazonian fish were highly correlated with ICP-MS. The squared correlation coefficient between TDA-AAS and ICP-MS for their results and ours was 0.96 and 0.97, respectively (Fig. 11) [62]. Additionally, the overall agreement between TDA-AAS and ICP-MS with a slightly elevated concentration obtained with TDA-AAS confirms what was recently shown when comparing the two techniques for analyzing marine sediment samples [63].

## Conclusions

We developed and thoroughly validated a non-chromatographic method for the determination of total mercury and methylmercury in finfish. Our results reaffirm TDA-AAS as a complementary technique to ICP-MS, providing quick and accurate results for total mercury in finfish at concentrations > 25 ng/g. Similarly, isolating methylmercury from the matrix prior to TDA-AAS detection is an efficient alternative to HPLC-ICP-MS. We have replaced toluene from legacy extraction methods with ethyl acetate, thereby using a greener and safer solvent. We demonstrate a SALLE extraction with NaCl that allows for emulsion-free phase separation of ethyl acetate from aqueous solutions of dilute HCl and Na_2_S_2_O_3_ from method development, including thorough investigations of methylmercury stability and selection bias. Featuring NaCl for phase partitioning allows for selective isolation of methylmercury from inorganic mercury with less than 7.5% false positive bias, and methylmercury extracts in 1 M NaCl and 100 mM Na_2_S_2_O_3_ are stable for up to 4 h on the instrument autosampler. This extraction method for methylmercury is accurate, efficient, and selective when coupled with mercury detection by TDA-AAS. From extraction to detection, accurate results were obtained from a sample in less than 2 h for both total mercury and methylmercury.

## Supplementary Information

Below is the link to the electronic supplementary material.

Supplementary file1 (DOCX 171 KB)

## Data Availability

All data is available upon reasonable request.

## References

[1] U.S. Food & Drug Administration (2024) Mercury in food. https://www.fda.gov/food/environmental-contaminants-food/mercury-food. Accessed 2024/12/14 2024.

[2] Fechner C, Hackethal C, Höpfner T, Dietrich J, Bloch D, Lindtner O, Sarvan I. Results of the BfR MEAL Study: in Germany, mercury is mostly contained in fish and seafood while cadmium, lead, and nickel are present in a broad spectrum of foods. Food Chemistry: X. 2022;14:100326. 35601214 10.1016/j.fochx.2022.100326 PMC9114524

[3] Médieu A, Point D, Sonke JE, Angot H, Allain V, Bodin N, Adams DH, Bignert A, Streets DG, Buchanan PB, Heimbürger-Boavida LE, Pethybridge H, Gillikin DP, Ménard F, Choy CA, Itai T, Bustamante P, Dhurmeea Z, Ferriss BE, Bourlès B, Habasque J, Verheyden A, Munaron JM, Laffont L, Gauthier O, Lorrain A. Stable Tuna mercury concentrations since 1971 illustrate marine inertia and the need for strong emission reductions under the Minamata Convention. Environ Sci Tech Let. 2024;11(3):250–8. 10.1021/acs.estlett.3c00949.

[4] Nogara PA, Oliveira CS, Schmitz GL, Piquini PC, Farina M, Aschner M. Methylmercury’s chemistry: from the environment to the mammalian brain. Biochimica et Biophysica Acta (BBA)-General Subjects. 1863;12:129284. 10.1016/j.bbagen.2019.01.006 30659885

[5] Gray PJ, Cunningham W. Inductively coupled plasma collision cell quadrupole mass spectrometric determination of extractible arsenic, cadmium, chromium, lead, mercury, and other elements in food using microwave-assisted digestion: results from an FDA interlaboratory study. J AOAC Int. 2019;102(2):590–604. 10.5740/jaoacint.18-0129. 30097073 10.5740/jaoacint.18-0129

[6] Montaser A. Inductively coupled plasma mass spectrometry. John Wiley & Sons; 1998.

[7] Nóbrega JA, Trevizan LC, Araújo GC, Nogueira ARA. Focused-microwave-assisted strategies for sample preparation. Spectrochim Acta, Part B. 2002;57(12):1855–76.

[8] Rocha DL, Batista AD, Rocha FR, Donati GL, Nobrega JA. Greening sample preparation in inorganic analysis. TrAC, Trends Anal Chem. 2013;45:79–92.

[9] Torres D, Martins-Teixeira M, Silva E, Queiroz H. Method development for the control determination of mercury in seafood by solid-sampling thermal decomposition amalgamation atomic absorption spectrometry (TDA AAS). Food Addit Contam: Part A. 2012;29(4):625–32. 10.1080/19440049.2011.642310 22214522

[10] Milestone The determination of total mercury in fish & biological tissues. https://www.milestonesci.com/wp-content/uploads/2021/01/AppRpt-DMA80evo-Fish_USREV061019.pdf. Accessed 2024/12/14 2024.

[11] Hight SC, Cheng J. Determination of methylmercury and estimation of total mercury in seafood using high performance liquid chromatography (HPLC) and inductively coupled plasma-mass spectrometry (ICP-MS): method development and validation. Anal Chim Acta. 2006;567(2):160–72. 10.1016/j.aca.2006.03.048.

[12] Scerbo R, Barghigiani C. Organic mercury determination in fish samples using an automatic mercury analyser. Environ Technol. 1998;19(3):339–42. 10.1080/09593331908616689.

[13] Yang HY, Jian R, Liao J, Cui J, Fang P, Zou ZR, Huang K. Recent development of non-chromatographic atomic spectrometry for speciation analysis of mercury. Appl Spectrosc Rev. 2022;57(6):441–60. 10.1080/05704928.2021.1893183.

[14] Westoo G. Determination of methylmercury compounds in foodstuffs .2. Determination of methylmercury in fish egg meat and liver. Acta Chem Scand. 1967;21(7):1790. 10.3891/acta.chem.scand.21-1790. 5625489 10.3891/acta.chem.scand.21-1790

[15] Westoo G. Determination of methylmercury compounds in foodstuffs .I. Methylmercury compounds in fish identification and determination. Acta Chem Scand. 1966;20(8):2131. 10.3891/acta.chem.scand.20-2131. 6007677 10.3891/acta.chem.scand.20-2131

[16] Capelli R, Fezia C, Franchi A, Zanicchi G. Extraction of methylmercury from fish and its determination by atomic-absorption spectroscopy. Analyst. 1979;104(1245):1197–200. 10.1039/an9790401197. 532980 10.1039/an9790401197

[17] Maggi C, Berducci MT, Bianchi J, Giani M, Campanella L. Methylmercury determination in marine sediment and organisms by direct mercury analyser. Anal Chim Acta. 2009;641(1–2):32–6. 10.1016/j.aca.2009.03.033. 19393363 10.1016/j.aca.2009.03.033

[18] Cordeiro F, Calderón J, Gonçalves S, Lourenço MH, Robouch P, Emteborg H, Conneely P, Tumba-Tshilumba MF, De la Calle MB. IMEP-115: determination of methylmercury in seafood by elemental mercury analysis: collaborative study. J AOAC Int. 2014;97(2):593–7. 10.5740/jaoacint.13-235. 24830172 10.5740/jaoacint.13-235

[19] Azemard S, Vassileva E. Determination of methylmercury in marine biota samples with advanced mercury analyzer: method validation. Food Chem. 2015;176:367–75. 10.1016/j.foodchem.2014.12.085. 25624245 10.1016/j.foodchem.2014.12.085

[20] Kim TH, Cho MJ, Lee Y, Kim JH, Hwang JY, Lee HE, Kim SH, Choi JD, Kang GJ. Methylmercury determination in fish by direct mercury analyzer. J AOAC Int. 2020;103(1):244–9. 10.5740/jaoacint.18-0254. 31537208 10.5740/jaoacint.18-0254

[21] Tobiszewski M, Namiesnik J, Pena-Pereira F. Environmental risk-based ranking of solvents using the combination of a multimedia model and multi-criteria decision analysis. Green Chem. 2017;19(4):1034–42. 10.1039/c6gc03424a.

[22] Alder CM, Hayler JD, Henderson RK, Redman AM, Shukla L, Shuster LE, Sneddon HF. Updating and further expanding GSK’s solvent sustainability guide. Green Chem. 2016;18(13):3879–90. 10.1039/c6gc00611f.

[23] Prat D, Wells A, Hayler J, Sneddon H, McElroy CR, Abou-Shehada S, Dunn PJ. CHEM21 selection guide of classical- and less classical-solvents. Green Chem. 2016;18(1):288–96. 10.1039/c5gc01008j.

[24] Byrne FP, Jin S, Paggiola G, Petchey TH, Clark JH, Farmer TJ, Hunt AJ, Robert McElroy C, Sherwood J. Tools and techniques for solvent selection: green solvent selection guides. SustaiN Chem Proc. 2016;4:1–24.

[25] Tang YQ, Weng N. Salting-out assisted liquid–liquid extraction for bioanalysis. Bioanalysis. 2013;5(12):1583–98. 23795935 10.4155/bio.13.117

[26] Matkovich CE, Christian GD. Salting-out of acetone from water - basis of a new solvent-extraction system. Anal Chem. 1973;45(11):1915–21. 10.1021/ac60333a023.

[27] Valente IM, Goncalves LM, Rodrigues JA. Another glimpse over the salting-out assisted liquid-liquid extraction in acetonitrile/water mixtures. J Chromatogr A. 2013;1308:58–62. 10.1016/j.chroma.2013.08.014. 23958692 10.1016/j.chroma.2013.08.014

[28] Li M, Zhuang B, Lu Y, An L, Wang ZG. Salt-induced liquid-liquid phase separation: combined experimental and theoretical investigation of water-acetonitrile-salt mixtures. J Am Chem Soc. 2021;143(2):773–84. 10.1021/jacs.0c09420. 33416302 10.1021/jacs.0c09420

[29] Zhao D-Y, Ding B, Zhu C-Y, Gong L, Duan F. Effects of inorganic salts on the phase separation of partially miscible solutes. Langmuir. 2024;40(11):5818–27. 38447182 10.1021/acs.langmuir.3c03693

[30] Young W, Wiggins S, Limm W, Fisher CM, DeJager L, Genualdi S. Analysis of per- and poly(fluoroalkyl) substances (PFASs) in highly consumed seafood products from U.S. markets. J Agric Food Chem. 2022;70(42):13545–53. 10.1021/acs.jafc.2c04673. 36251396 10.1021/acs.jafc.2c04673 PMC9614959

[31] U.S. Food and Drug Administration. Elemental Analysis Manual 4.7: Inductively coupled plasma-mass spectrometric determination of arsenic, cadmium, chromium, lead, mercury, and other elements in food using microwave assisted digestion. 2020. https://www.fda.gov/media/87509/download. Accessed 2025/07/03. 10.5740/jaoacint.18-0129 30097073

[32] U.S. Food and Drug Administration. Elemental Analysis Manual for food and related products 4.8: High performance liquid chromatographic inductively coupled plasma-mass spectrometric determination of methylmercury and total mercury in seafood. 2008. https://www.fda.gov/media/95174/download. Accessed 2025/07/03.

[33] U.S. EPA. Method 7473 (SW-846): Mercury in solids and solutions by thermal decomposition, amalgamation, and atomic absorption spectrophotometry. 1998. https://www.epa.gov/sites/default/files/2015-07/documents/epa-7473.pdf. Accessed 2025/07/03.

[34] Rahman GMM, Kingston HM. Development of a microwave-assisted extraction method and isotopic validation of mercury species in soils and sediments. J Anal Atom Spectrom. 2005;20(3):183–91. 10.1039/b404581e.

[35] U.S. Food and Drug Administration. Elemental Analysis Manual for food and related products 3.2: Terminology. 2021. https://www.fda.gov/media/89337/download. Accessed 2025/07/05.

[36] Jagtap R, Krikowa F, Maher W, Foster S, Ellwood M. Measurement of methyl mercury (I) and mercury (II) in fish tissues and sediments by HPLC-ICPMS and HPLC-HGAAS. Talanta. 2011;85(1):49–55. 10.1016/j.talanta.2011.03.022. 21645668 10.1016/j.talanta.2011.03.022

[37] Rai R, Maher W, Kirkowa F. Measurement of inorganic and methylmercury in fish tissues by enzymatic hydrolysis and HPLC-ICP-MS. J Anal Atom Spectrom. 2002;17(11):1560–3. 10.1039/b208041a.

[38] MacCrehan WA, Durst RA. Measurement of organomercury species in biological samples by liquid chromatography with differential pulse electrochemical detection. Anal Chem. 1978;50(14):2108–12. 10.1021/ac50036a040. 569449 10.1021/ac50036a040

[39] International Union of Pure and Applied Chemistry. The international harmonized protocol for the proficiency testing of (chemical) analytical laboratories. Pure Appl Chem. 1993;65(9):2123–44.

[40] Rosera TJ, Janssen SE, Tate MT, Lepak RF, Ogorek JM, DeWild JF, Babiarz CL, Krabbenhoft DP, Hurley JP. Isolation of methylmercury using distillation and anion-exchange chromatography for isotopic analyses in natural matrices. Anal Bioanal Chem. 2020;412(3):681–90. 10.1007/s00216-019-02277-0. 31834449 10.1007/s00216-019-02277-0

[41] Powell KJ, Brown PL, Byrne RH, Gajda T, Hefter G, Sjöberg S, Wanner H. Chemical speciation of environmentally significant heavy metals with inorganic ligands. Part 1: The Hg2+–Cl–, OH–, CO32–, SO42–, and PO43–aqueous systems (IUPAC Technical Report). Pure Appl Chem. 2005;77(4):739–800.

[42] Sanz J, Raposo JC, Larreta J, Martinez-Arkarazo I, de Diego A, Madariaga JM. On-line separation for the speciation of mercury in natural waters by flow injection-cold vapour-atomic absorption spectrometry. J Sep Sci. 2004;27(14):1202–10. 10.1002/jssc.200301701. 15537077 10.1002/jssc.200301701

[43] Liem-Nguyen V, Skyllberg U, Bjorn E. Thermodynamic modeling of the solubility and chemical speciation of mercury and methylmercury driven by organic thiols and micromolar sulfide concentrations in boreal wetland soils. Environ Sci Technol. 2017;51(7):3678–86. 28248107 10.1021/acs.est.6b04622

[44] Manceau A, Lemouchi C, Enescu M, Gaillot A-C, Lanson M, Magnin V, Glatzel P, Poulin BA, Ryan JN, Aiken GR. Formation of mercury sulfide from Hg (II)–thiolate complexes in natural organic matter. Environ Sci Technol. 2015;49(16):9787–96. 26168020 10.1021/acs.est.5b02522

[45] Schwarzenbach G, Schellenberg M. Die komplexchemie des methylquecksilber-kations. Helv Chim Acta. 1965;48(1):28–46.

[46] Westoo G. Determination of methylmercury salts in various kinds of biological material. Acta Chem Scand. 1968;22(7):2277–3000. 10.3891/acta.chem.scand.22-2277. 5751873 10.3891/acta.chem.scand.22-2277

[47] Hyde AM, Zultanski SL, Waldman JH, Zhong YL, Shevlin M, Peng F. General principles and strategies for salting-out informed by the Hofmeister series. Org Process Res Dev. 2017;21(9):1355–70. 10.1021/acs.oprd.7b00197.

[48] Tobias DJ, Hemminger JC. Chemistry - getting specific about specific ion effects. Science. 2008;319(5867):1197–8. 10.1126/science.1152799. 18309069 10.1126/science.1152799

[49] Fernandez-Bautista T, Gomez-Gomez B, Gracia-Lor E, Perez-Corona T, Madrid Y. Selenium health benefit values and Hg and Se speciation studies for elucidating the quality and safety of highly consumed fish species and fish-derived products. Food Chem. 2024;435:137544. 10.1016/j.foodchem.2023.137544. 37774614 10.1016/j.foodchem.2023.137544

[50] Yu LP, Yan XP. Factors affecting the stability of inorganic and methylmercury during sample storage. Trac-Trend Anal Chem. 2003;22(4):245–53. 10.1016/S0165-9936(03)00407-2.

[51] Reyes LH, Mizanur Rahman GM, Fahrenholz T, Skip Kingston HM. Comparison of methods with respect to efficiencies, recoveries, and quantitation of mercury species interconversions in food demonstrated using tuna fish. Anal Bioanal Chem. 2008;390(8):2123–32. 10.1007/s00216-008-1966-3. 18317738 10.1007/s00216-008-1966-3

[52] White TL, Brown LW, Looney BB, Jones MA. Total mercury analysis comparison: Deployment of analytical method for the Savannah River Site Liquid Waste System. Savannah River National Laboratory. 2019. https://sti.srs.gov/fulltext/SRNL-STI-2019-00056.pdf. Accessed 2025/07/03.

[53] Gajdosechova Z, Lawan MM, Urgast DS, Raab A, Scheckel KG, Lombi E, Kopittke PM, Loeschner K, Larsen EH, Woods G. In vivo formation of natural HgSe nanoparticles in the liver and brain of pilot whales. Sci Rep-Uk. 2016;6(1):34361. 10.1038/srep34361 PMC5039623 27678068

[54] Manceau A, Gaillot A-C, Glatzel P, Cherel Y, Bustamante P. In vivo formation of HgSe nanoparticles and Hg–tetraselenolate complex from methylmercury in seabirds—implications for the Hg–Se antagonism. Environ Sci Technol. 2021;55(3):1515–26. 33476140 10.1021/acs.est.0c06269

[55] U.S. Food and Drug Administration Foods Program. Guidelines for the validation of chemical methods in food, feed, cosmetics, and veterinary products. 3rd ed. 2019. https://www.fda.gov/media/81810/download?attachment. Accessed 2025/07/03.

[56] Floreto EA, Prince DL, Brown PB, Bayer RC. The biochemical profiles of shell-diseased American lobsters Homarus americanus Milne Edwards. Aquaculture. 2000;188(3–4):247–62.

[57] Favilli L, Giacomino A, Malandrino M, Inaudi P, Diana A, Abollino O. Strategies for mercury speciation with single and multi-element approaches by HPLC-ICP-MS. Front Chem. 2022;10:1082956. 10.3389/fchem.2022.1082956. 36531326 10.3389/fchem.2022.1082956 PMC9754325

[58] Amde M, Yin Y, Zhang D, Liu J. Methods and recent advances in speciation analysis of mercury chemical species in environmental samples: a review. Chem Speciat Bioavailab. 2016;28(1–4):51–65.

[59] Crowther ER, Demers JD, Blum JD, Brooks SC, Johnson MW. Coupling of nitric acid digestion and anion-exchange resin separation for the determination of methylmercury isotopic composition within organisms. Anal Bioanal Chem. 2023;415(5):759–74. 10.1007/s00216-022-04468-8. 36472636 10.1007/s00216-022-04468-8

[60] Taylor VF, Carter A, Davies C, Jackson BP. Trace-level automated mercury speciation analysis. Anal Methods. 2011;3(5):1143–8. 10.1039/C0AY00528B. 21572543 10.1039/C0AY00528B PMC3092719

[61] Jackson B, Taylor V, Baker RA, Miller E. Low-level mercury speciation in freshwaters by isotope dilution GC-ICP-MS. Environ Sci Technol. 2009;43(7):2463–9. 19452902 10.1021/es802656p PMC2692077

[62] Neto OGN, Dias SR, Albuquerque FEA, Miranda M, Lopez-Alonso M, Oliveira RB, Pinto D, Minervino AHH. Comparative analysis between mercury levels in fish tissues evaluated using direct mercury analyzer and inductively plasma-coupled mass spectrometer. Chemosphere. 2024;351:141146. 38211792 10.1016/j.chemosphere.2024.141146

[63] Provete CS, Dalfior BM, Mantovaneli R, Carneiro MTW, Brandão GP. Comparison of the performance of ICP-MS, CV-ICP-OES, and TDA AAS in determining mercury in marine sediment samples. ACS Omega. 2024;9(50):49229–38. 39713621 10.1021/acsomega.4c06144 PMC11656208

[64] Passing H, Bablok. A new biometrical procedure for testing the equality of measurements from two different analytical methods. Application of linear regression procedures for method comparison studies in clinical chemistry, Part I. J Clin Chem Clin Biochem. 1983;21(11):709–20. 10.1515/cclm.1983.21.11.709. 6655447 10.1515/cclm.1983.21.11.709

