# Supplementary material for: Development and validation of a non-chromatographic method for mercury and methylmercury in finfish using thermal decomposition gold amalgamation atomic absorption spectrophotometry (TDA-AAS) and salting-out assisted liquid–liquid extraction (SALLE)
Source: Anal Bioanal Chem. 2025 Jul 16;417(20):4723–38. doi: 10.1007/s00216-025-05989-8 (PMC12325422; doi:10.1007/s00216-025-05989-8)

**Supplementary Information**

**Table S1.** Sample information [30].

**Table S2.** Summary statistics from reference material analyses using the optimized methylmercury method. Certificate and uncertainty values listed for methylmercury.

**Table S3.** Summary statistics for total mercury from reference material analyses. Certificate and uncertainty values listed for total mercury. These results are from calibrating the instrument with liquid mercury standards, not reference materials.

**Figure S1 (double column width, 174 mm).** Results from methylmercury analyses of reference materials. (a) Recoveries compared to reference material uncertainty. The solid horizontal line is the 95% uncertainty range for the reference material. The solid vertical lines represent the 80—110% recovery range. (b) Z scores from reference material analyses summarized as a boxplot and point overlay.

**Table S1.**

| **Sample** | **Item Description** | **Wild-caught (W) vs. Aquaculture (A)** | **Country of Origin** | **Packaging** |
| --- | --- | --- | --- | --- |
| Clams 1-1 | Smoked baby clams in soybean oil | NI | China | Can |
| Clams 1-2 | Smoked baby clams in cottonseed oil | A | China | Can |
| Clams 1-3 | Smoked clams in cottonseed oil | NI | China | Can |
| Clams 1-6 | Smoked baby clams in soybean oil | NI | China | Can |
| Clams 1-7 | Whole baby clams in water | NI | China | Can |
| Clams 1-8 | Chopped baby clams in water | NI | China | Can |
| Clams 1-9 | Whole baby clams in water | W | China | Can |
| Cod 1-1 | Cod | W | China | LDPE |
| Cod 1-2 | Cod loin | NI | NI | LDPE |
| Cod 1-3 | Fillets | W | Iceland | LDPE |
| Cod 1-4 | Pacific cod | W | China | LDPE |
| Cod 1-5 | Cod | W | China | LDPE |
| Cod 1-6 | Pacific cod | W | China* (United States) | LDPE |
| Cod 1-7 | Pacific cod | W | China | LDPE |
| Cod 1-8 | Atlantic cod | W | Iceland | not available |
| Cod 1-9 | Atlantic cod | W | Norway | LDPE |
| Pollock 1-1 | Breaded fillets | W | NI | LDPE |
| Pollock 1-10 | Fillets | W | China* (United States) | LDPE |
| Pollock 1-2 | Fish sticks | W | NI | LDPE |
| Pollock 1-3 | Fish sticks | W | Germany | LDPE |
| Pollock 1-4 | Fillets | W | United States | Paper |
| Pollock 1-5 | Panko breaded fish sticks | W | United States | LDPE |
| Pollock 1-6 | Fish sticks | W | United States | PP |
| Pollock 1-7 | Fish sticks | W | United States | LDPE |
| Pollock 1-8 | Beer battered | W | United States | LDPE |
| Pollock 1-9 | Salted pollock | W | China | LDPE |
| Salmon 1-1 | Atlantic salmon | A | Chile | LDPE |
| Salmon 1-2 | Atlantic salmon | A | Chile | HDPE |
| Salmon 1-3 | Atlantic salmon | A | Chile | HDPE |
| Salmon 1-4 | Atlantic salmon | A | Norway | PET |
| Salmon 1-5 | Atlantic salmon | A | Canada | Expanded PS |
| Salmon 1-6 | Atlantic salmon | A | Norway | LDPE |
| Salmon 1-7 | Atlantic salmon | A | Chile | LDPE |
| Salmon 1-8 | Atlantic salmon | A | Norway | PE/PVA |
| Salmon 1-9 | Atlantic salmon | A | Norway | Paper |
| Shrimp 1-10 | 16-20 count shrimp | A | Indonesia | LDPE |
| Shrimp 1-2 | 26-30 count shrimp | A | NI | LDPE |
| Shrimp 1-3 | 41-50 count shrimp | A | India | LDPE |
| Shrimp 1-5 | 16-20 count shrimp | A | Indonesia | LDPE |
| Shrimp 1-6 | 21-25 count shrimp | A | India | LDPE |
| Shrimp 1-7 | 41-50 count shrimp | A | Indonesia | LDPE |
| Shrimp 1-8 | 31-40 count shrimp | A | Indonesia | LDPE |
| Shrimp 1-9 | 21-25 count shrimp | A | Indonesia | LDPE |
| Tilapia 1-1 | Tilapia fillets | A | China | LDPE |
| Tilapia 1-10 | Tilapia fillets | A | China | LDPE |
| Tilapia 1-2 | Tilapia fillets | A | China | LDPE |
| Tilapia 1-3 | Tilapia fillets | A | Honduras | LDPE |
| Tilapia 1-4 | Tilapia fillets | A | NI | LDPE |
| Tilapia 1-5 | Tilapia fillets | A | China | LDPE |
| Tilapia 1-6 | Tilapia fillets | A | China | LDPE |
| Tilapia 1-7 | Tilapia fillets | A | China | LDPE |
| Tilapia 1-8 | Tilapia fillets | A | NI | LDPE |
| Tilapia 1-9 | Tilapia fillets | NI | NI | Expanded PS |
| Tuna 035 | Yellowfin tuna fillets with garlic in olive oil | W | Costa Rica | Glass jar |
| Tuna 036 | Solid white albacore tuna in water | W | NI | Can |
| Tuna 1-1 | Lemon pepper tuna | W | Ecuador | Foil pouch |
| Tuna 1-10 | Chunk light tuna in water | W | NI | Can |
| Tuna 1-2 | Chunk light tuna in water | W | Thailand | Can |
| Tuna 1-3 | Chunk light tuna in water | W | Thailand | Can |
| Tuna 1-4 | Chunk white albacore tuna in water | W | NI | Can |
| Tuna 1-5 | Solid white albacore tuna in extra virgin olive oil | W | NI | Can |
| Tuna 1-6 | Chunk light tuna in water | W | Thailand | Can |
| Tuna 1-8 | Chunk white albacore tuna in water | W | Thailand | Can |
| Tuna 1-9 | Solid white albacore tuna in water | W | NI | Can |
| Tuna 10 | NA | NA | NA | NA |
| Crab 1-1 | Claw meat | W | Indonesia | Can |
| Crab 1-10 | Blue swimming crabmeat jumbo lump | W | Indonesia | PP |
| Crab 1-2 | Crab claw | W | Indonesia | PP |
| Crab 1-3 | Crab jumbo | W | Indonesia | PP |
| Crab 1-4 | Crab claw | W | Indonesia | PP |
| Crab 1-5 | Special claw blend | W | Indonesia | PP |
| Crab 1-6 | Blue crab claw fingers | W | Mexico | Can |
| Crab 1-7 | Crab lump | W | Indonesia | PP |
| Crab 1-8 | Blue crab cocktail claws | W | Mexico | Can |
| Crab 1-9 | Blue swimming crabmeat lump | W | Indonesia | PP |

*Indicates the country where the product was processed; however, the label indicated a different country of harvest. NI = not indicated on the label. NA = not available.

**Table S2.**

| **Reference Material** | **Mean Recovery ± 2σ (%)** | **Mean Concentration ± 2σ (ng/g)** | **Certificate Value ± 95% uncertainty (ng/g)** | **Z score** | **n** |
| --- | --- | --- | --- | --- | --- |
| NIST SRM 1947 Lake Michigan Fish Tissue | 105 ± 12 | 244 ± 28 | 233 ± 10 | -1.03 — 1.66 | 8 |
| NIST SRM 2976 Trace Elements and Methylmercury in Mussel Tissue (Freeze-Dried) | 104 ± 11 | 29 ± 3 | 28.09 ± 0.31 | -1.19 — 1.55 | 8 |
| IAEA 436a Tuna Fish Flesh Homogenate | 94 ± 7 | 3386 ± 239 | 3620 ± 470 | -1.98 — 0.32 | 20 |
| IAEA 476 Fish Homogenate | 91 ± 6 | 477 ± 32 | 523 ± 30 | -1.92 — 0.29 | 17 |
| NRC DOLT-3 Dogfish Liver | 86 ± 1 | 1365 ± 22 | 1590 ± 120 | -1.68 — -1.34 | 3 |
| NRC DORM-3 Fish Protein | 80 ± 1 | 284 ± 5 | 355 ± 56 | -1.95 — -1.69 | 3 |
| NIST RM-50 Albacore Tuna | 94 ± 4 | 829 ± 35 | 880 ± 70 | -1.59 — -0.08 | 23 |

**Table S3.**

| **Reference Material** | **Mean Recovery +/- 2σ (%)** | **Mean Concentration +/- 2σ (ng/g)** | **Certificate Value +/- 95% uncertainty (ng/g)** | **Z score range** | **n** |
| --- | --- | --- | --- | --- | --- |
| NIST SRM 1566b Oyster Tissue | 101 +/- 6 | 38 +/- 2 | 37.1 +/- 1.3 | -0.45 — 0.56 | 17 |
| NIST SRM 2976 Trace Elements and Methylmercury in Mussel Tissue (Freeze-Dried) | 116 +/- 11 | 71 +/- 7 | 61 +/- 3.6 | 0.15 — 1.95 | 17 |
| IAEA 436a Tuna Fish Flesh Homogenate | 101 +/- 19 | 4311 +/- 801 | 4260 +/- 360 | -0.66 — 2.75 | 21 |
| IAEA 476 Fish Homogenate | 103 +/- 14 | 596 +/- 81 | 578 +/- 22 | -0.3 — 1.46 | 10 |
| NRC DOLT-5 Dogfish Liver | 96 +/- 27 | 424 +/- 118 | 440 +/- 180 | -0.88 — 1.37 | 24 |
| NIST RM-50 Albacore Tuna | 118 +/- 29 | 1121 +/- 278 | 950 +/- 100 | 0.11 — 2.35 | 7 |
| NRC TORT-3 Lobster Hepatopancreas | 103 +/- 9 | 300 +/- 28 | 292 +/- 22 | -0.24 — 0.92 | 6 |

**Figure S1.**


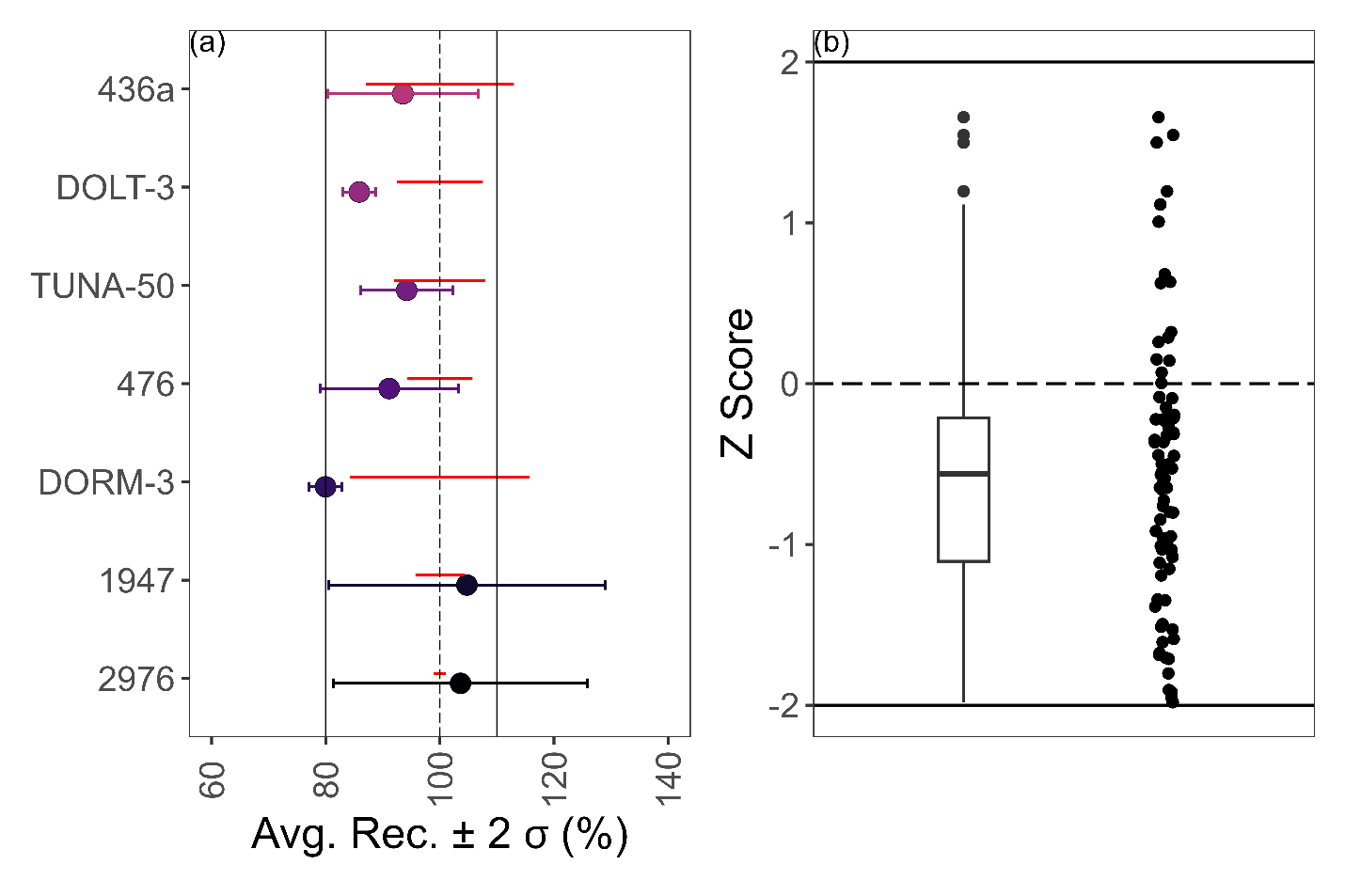

Supplement: Supplementary file 1 — Supplementary file1 (DOCX 171 KB) [file 216_2025_5989_MOESM1_ESM.docx]
